# Identification of quantitative trait loci controlling soybean seed protein and oil content

**DOI:** 10.1371/journal.pone.0286329

**Published:** 2023-06-23

**Authors:** Elizabeth M. Clevinger, Ruslan Biyashev, David Haak, Qijian Song, Guillaume Pilot, M. A. Saghai Maroof

**Affiliations:** 1 School of Plant and Environmental Sciences, Virginia Tech, Blacksburg, Virginia, United States of America; 2 Soybean Genomics and Improvement Lab, United States Department of Agriculture-Agricultural Research Service, Beltsville, Maryland, United States of America; Jeju National University, REPUBLIC OF KOREA

## Abstract

Soybean is a major source of seed protein and oil globally with an average composition of 40% protein and 20% oil in the seed. The goal of this study was to identify quantitative trait loci (QTL) conferring seed protein and oil content utilizing a population constructed by crossing an above average protein content line, PI 399084 to another line that had a low protein content value, PI 507429, both from the USDA soybean germplasm collection. The recombinant inbred line (RIL) population, PI 507429 x PI 399084, was evaluated in two replications over four years (2018–2021); the seeds were analyzed for seed protein and oil content using near-infrared reflectance spectroscopy. The recombinant inbred lines and the two parents were re-sequenced using genotyping by sequencing. A total of 12,761 molecular markers, which came from genotyping by sequencing, the SoySNP6k BeadChip and selected simple sequence repeat (SSR) markers from known protein QTL chromosomal regions were used for mapping. One QTL was identified on chromosome 2 explaining up to 56.8% of the variation for seed protein content and up to 43% for seed oil content. Another QTL identified on chromosome 15 explained up to 27.2% of the variation for seed protein and up to 41% of the variation for seed oil content. The protein and oil QTLs of this study and their associated molecular markers will be useful in breeding to improve nutritional quality in soybean.

## Introduction

Soybean (*Glycine max* (L.) Merr.) is one of the major sources of seed protein and oil in the United States and around the world with an average composition of 40% protein and 20% oil [1]. It is also a source for essential amino acids and metabolizable energy for both human and animal consumption [2]. One of the most important uses of soybean is protein rich soybean meal for poultry and swine feed, since it has the highest level of crude protein among plant-based protein sources [3, 4]. Conventional cultivars of soybean generally have protein values between 38–42% on a dry weight basis in the seed. The U.S. used 33.5 million metric tons of soybean meal in 2021 for livestock feed with the majority going to poultry. The U.S. also consumed 10.6 million metric tons of soybean oil in 2021 (http://soystats.com). Increasing protein and oil content in soybean seeds would enhance the economic value for growers and processors [5]. Soybean seed protein and oil content are complex quantitatively inherited polygenic traits [6–8]. Increasing protein content is problematic due to the negative correlation with oil content, carbohydrates and seed yield and is affected by environmental conditions [9–14]. It has been thoroughly documented that there is a negative correlation between oil and protein, typically a 1% reduction in total oil content will lead to a 2% increase in total protein content [14–16]. It has also been reported that soybean protein content is higher and oil content lower in the Southeast United States compared to the Midwest [15].

Quantitative trait loci (QTL) controlling protein and oil concentrations have been mapped to all 20 soybean chromosomes. Over 250 protein QTL from bi-parental population studies are currently listed in Soybase (http://soybase.org, 2021). QTLs for protein and oil content have been repeatedly found and mapped on chromosomes 15 and 20. Diers et al. [17] first mapped protein and oil QTL to chromosomes 15 and 20 using a cross developed from an experimental *G*. *max* line and a *G*. *soja* accession. Chung et al. [13] using 76 recombinant inbred lines (RILs) derived from a high protein by high yield cross was able to determine that the same chromosome 20 QTL detected in other populations was also segregating in this population. Wang et al. [18] identified and validated QTLs associated with seed yield, protein and oil content in two different RIL populations. They were able to identify QTLs for protein and oil content on chromosomes 2, 3, 4, 9, 11, 17 and 20. Bandillo et al. [19], conducted genome wide association studies (GWAS) using 12,000 accessions from the USDA germplasm collection to detect seed protein and oil QTL. This study identified significant single nucleotide polymorphisms (SNPs) associated with both seed protein and oil content on both chromosomes 15 and 20. The identified chromosome 20 region was in the same location as those that had been reported in previous studies and was further narrowed with this study. Warrington et al. [20] were able to identify seed protein QTL on chromosomes 14, 15, 17 and 20 and the QTL found on chromosome 20 explained 55% of the phenotypic variation in their population. Phansak et al. [1] used selective genotyping-based QTL analysis to survey 48 populations to observe both previously known and unknown protein QTL. Lee et al. [21] conducted a genome-wide association study utilizing data from five environments for over six hundred accessions from multiple maturity groups. They identified QTL for seed protein, oil and amino acid content. Significant SNPs for seed protein and oil content were found on chromosomes 15, 19 and 20 in this study. Zhang et al. [22] used linkage analysis and GWAS on over 300 RILs and 200 soybean accessions to identify 15 QTLs affecting protein and/or oil content. QTL regions for seed protein and oil content have now been very well researched especially those meta-QTL on chromosomes 15 and 20 [23]. As these authors note these loci may be used for improvement but for just one trait at a time since there is such a strong negative correlation between protein and oil content associated with these loci.

Recently, progress has been made on discovery of the genes that may be responsible for protein content on chromosomes 15 and 20. Patil et al. [24] identified 52 putative SWEET genes, that play various roles within plants. For example, *Glyma*.*15G049200* is a sucrose efflux transporter gene and is highly expressed in soybean seeds and leaves [25]. Using a combination of RNA-seq data and qRT-PCR comparing two soybean accessions differing in oil content they showed that the *Glyma*.*15G049200* gene was positively correlated with seed oil content and has been selected to increase the oil content in soybean breeding. Zhang et al. [26] using association analysis identified an insertion/deletion (—/CC) within *Glyma*.*15G049200*, which was associated with both protein and oil content. The CC deletion caused the deletion of 19 amino acids, a premature stop codon and six amino acid changes from the C-terminus of *Glyma*.*15G049200* in Williams82. Three amino acids were also changed in the cytoplasmic C-terminal tail that appear to be highly conserved in legumes. It is undetermined though how these changes affect the activity of *Glyma*.*15G049200* and how this impacts oil and protein content [26]. They observed that the accessions in this study with the presence of CC (CC+) contained 9.5% less oil and 4.6% more protein than those with the CC deletion (CC-). Their findings suggest that the two alleles CC+ and CC- may be used for developing high protein lines or for oil improvement, respectively [26]. Fliege et al. [27] were able to fine map the cqSeed protein-003 QTL on chromosome 20 that has the greatest additive effect for seed protein content. Through fine mapping and positional cloning, they identified an insertion/deletion polymorphism in *Glyma*.*20G085100* that controlled seed protein. Goettel et al. [28] identified a transposable element insertion within *Glyma*.*20G085100* that causes significant increases in seed oil content and weight with a decrease in protein content.

Identification of unique QTLs responsible for high protein content is a critical step in the development of high protein content soybean cultivars. The USDA soybean germplasm collection has been reported to have phenotypic variation for protein content ranging from 34.1% to 56.8% [29]. The goal of this study was to identify QTL associated with seed protein and oil content. For this purpose, a population was constructed by crossing a high protein content line with a low protein content line, both from the USDA soybean germplasm collection. This segregating population allowed the identification of large effect QTLs for protein and oil content on chromosome 2 and chromosome 15.

## Materials and methods

### Genetic material

The population used in this study consisted of 96 recombinant inbred lines (RILs) from the cross PI507429 x PI399084. Plant Introduction (PI) 507429 (Tousan 89) was chosen based on its low protein content from the germplasm resource information network (GRIN) from the USDA. PI 399084 (Chungchong Namdo) was chosen based on a higher than average protein content based on the GRIN database. These two plant introduction lines were crossed in the summer of 2010 and advanced annually in the field in Blacksburg, Virginia (37°11ʹ53.15˝N, 80°34ʹ24.77˝W), via the single seed descent method. This RIL population (F_7_-F_10_) was planted over four years in 2018–2021, in two replications each year. The average of the parental lines over these four years for seed protein and oil content were 39.7%, 14.2% and 30.9%, 18.9%, for PI 399084 and PI 507429, respectively.

Young first or second trifoliate leaves of greenhouse-grown F_7-9_ plants were collected for DNA extraction. DNA from parental lines and at least 10 bulked plants from each individual RIL was isolated from lyophilized tissues using the CTAB method as described in Saghai Maroof et al. [30] with minor modifications. DNA concentration was measured with a DyNA Quanta2000 Fluorometer (Hoefer®Scientific, San Francisco, CA).

### Phenotypic analysis

The seeds were analyzed for protein and oil content using near-infrared reflectance spectroscopy (NIRS) utilizing a DA 7250 NIR Analyzer (Perten Instruments, Springfield, IL). Briefly, around 42 g of seed from each line were put in a clear 2 oz. plastic cup and were analyzed for protein and oil content on a dry basis percentage and converted to a 13% moisture basis. Samples were measured 3 times and then averaged for each sample. Protein content was also independently determined by combustion on replications 1 and 2 from 2020 to confirm results obtained from NIRS analysis of those same replications/year (Eurofins Scientific Inc., Des Moines, Iowa, [AOAC 992.15, AOAC 990.03 and AOCS Ba4e-93]).

### Statistical analysis

R version 3.6.1 was used to perform single-factor ANOVA and variance component analysis using the protein and oil data from the RIL population. The estimated variance components were used to compute the broad sense heritability (*H*^*2*^) for seed protein and oil based on the data from the 2 replications of 2018–2021 plantings. Broad sense heritability (*H*^*2*^) was calculated as:

$$H^{2}=\sigma_{G}^{2}/[\sigma_{G}^{2}+\left(\frac{\sigma_{GE}^{2}}{e}\right)+\left(\frac{\sigma^{2}}{re}\right)]$$

in which $\sigma_{G}^{2}$ and $\sigma_{GE}^{2}$ refer to genotypic variance and genotype x environment variance, respectively. Coefficients *e* and *r* refer to the number of environments and replications within environments [31].

Correlations between protein and oil content for the two replications from each year from 2018 to 2021 and the averages of those replications for each year were calculated using R version 3.6.1

### Genotyping by sequencing

A total of 96 recombinant inbred lines (RILs) and two parents were sequenced using genotyping by sequencing (GBS). The parents were also subjected to high-depth resequencing (>50X) to generate a high-density panel of markers. Genomic DNA was extracted from leaf tissue using the CTAB method as described in Saghai Maroof et al. [30] with minor modifications. These DNA samples were digested and prepared for as libraries for genotyping by sequencing as described in the protocol by [32]. Briefly, genomic DNA templates from 102 individuals were digested with ApeKI 6-base cutter (GCWGC) to reduce the genome complexity. Two 51-plex GBS libraries comprising 102 total DNA from 96 RILs, 2 parents replicated 2 times and 2 bulk samples, were prepared by ligating the digested DNA to unique barcode nucleotide adapters, followed by standard Polymerase Chain Reaction (PCR). The resulting 51-plex libraries were sequenced in two lanes of Illumina NovaSeq 6000 2 x 150bp. In addition, standard DNAseq libraries were constructed for each of the PI parents (N = 2) and sequenced using a single lane of Illumina NovaSeq 6000.

### Sequencing data analysis and SNP calling

Raw sequence data were aligned to the soybean reference genome (*Glycine max* Wm82.a4.v1); [33] using bwa with the MEM option (version 0.7.17; [34]. Alignment files were sorted and PCR duplicates removed using samtools (version 1.2.1). Sorted bam files were simultaneously analyzed using freebayes (version-1.0; [35] with the following settings,—strict-vcf,—genotype-qualities,—pooled-continuous, -F 0.1 -C 5. This output a single vcf file with raw SNPs which were then filtered following the dDocent guidelines [36]. In short, using vcftools [37] variants were filtered for depth > 5, quality > Q30, and initially 50% missingness. This file was used to screen samples for high levels of missingness (all were <30%). The final SNP set was filtered for a maximum of 15% missing values and a minor allele frequency < 0.05, and 26 samples were removed due to missingness. These SNP markers were designated as GBS followed by assigned chromosome number and physical position (bp value) based on W82.a4.v1 assembly.

### Molecular marker assay

To identify chromosomal regions associated with protein and oil content, the segregating population was genotyped using the Illumina Infinium SoySNP6K BeadChip [38] in addition to the SNPs identified through GBS. SNPs in the 6k BeadChip were selected from the SoySNP50K [39]. The 6K marker data set was processed using GenomeStudio software (version 3.2.23). SNP markers that were monomorphic between parents of the RIL population and those which had more than 20% missing data were not used for linkage map construction. To relate our results to previous studies, a set of 26 Simple Sequence Repeat (SSR) markers from the known protein QTL chromosomal regions on chromosomes 2 and 15 were also mapped in the RIL population. Several GBS SNPs from the protein and oil QTL regions of this study were converted to KASP markers (GBS based SNPs designated with “K” at the end of the corresponding GBS loci). Primer sequences of KASP SNP markers are presented in the S1 Table. SSR markers were amplified by PCR with dye labeled forward primers [40] and analyzed by capillary electrophoresis using an Applied Biosystems 3130xl Genetic Analyzer unit (Carlsbad, CA, USA).

### Map construction and quantitative trait locus analysis

SNP data from the GBS and SoySNP6K of the RIL population were used for linkage map construction. JoinMap 4.0 [41] with a LOD threshold of 4.0 and a maximum recombination frequency of 50% for the original grouping was employed. Marker order and their positions within each linkage group were determined using the maximum likelihood algorithm and Kosambi mapping function; those unassigned to any linkage group (LG) were excluded.

MapQTL 5.0 software [42] was used for the identification of oil and protein QTL. Each round of QTL analysis was performed in two stages: Interval Mapping (IM) to detect critical chromosomal regions followed by more detailed QTL mapping provided by the enhanced power of multiple-QTL (MQM) method with the walking speed set to 1.0 cM.

In order to identify levels of LOD significance thresholds on both genome-wide and individual chromosome basis, permutation tests were conducted over 1000 iterations. A genome-wide LOD threshold was calculated at 3.5 and was used as a base line for QTL justification.

### CC-/CC+ sequencing

Using the genomic sequence of Williams 82 *Glyma*.*15g49200*, a pair of primers were designed to encompass CC indel in exon 6 of the gene, which is associated with seed protein/oil content as reported by Zhang et al. [26]. Primer design software within DNASTAR Lasergene 17 package was used for appropriate oligo-pairs selection. The designed primer sequences TGGGGCTAGTTCAGATGGT (Forward) and AATTGATACTCCATTGAGGTAGT (Reverse) were used for PCR amplification and Sanger (regular) sequencing. PCR product sizes were 340 bp with CC deletion and 342 bp with insertion. Sequencing was conducted by Eton Biosciences Inc. (Research Triangle Park, NC). It was determined that the parental lines, PI 507429 and PI 399084 were polymorphic for this indel and therefore, all RILs were sequence-genotyped for this indel.

## Results

### Phenotypic and statistical analyses

The population was planted over four years, (2018–2021), with two replications each year. The data from each replication and the mean of those two replications resulted in twelve data sets using NIRS for protein and oil assay. In addition to NIRS, seeds from both replications in 2020 were assayed via protein combustion by Eurofins (Des Moines, IA); the mean of these two replications resulted in three data sets. QTL mapping was conducted using a total of 15 protein data sets including: 12 data sets based on NIRS assay and three data sets based on the protein combustion assay. The range of protein content values for all 15 data sets varies from a low of 26.84% in 2021 Rep 2 to a high of 46.59% in protein combustion 2020 Rep1 (Table 1). The skewness for all protein content data sets was negative and varied between moderately skewed to symmetric (Table 1). The range of oil content values for the 12 data sets was quite variable within the population from a low of 11.09% in the second replication of 2019 to a high of 20.74% in the first replication from 2020 (Table 2). The skewness of the oil content data set is symmetric for all of the data sets.

**Table 1:** Descriptive statistics of the 15 protein content data sets used to identify QTL on chromosomes 2 and 15 in the RIL population of PI 507429 x PI 399084.

Variable	Range	Mean	Std Dev	Skewness	Kurtosis
2018 Rep1	31.41–42.93	37.34	2.65	-0.40	-0.70
2018 Rep2	31.47–43.23	37.73	2.40	-0.34	-0.19
2018 Avg.	32.10–42.46	37.52	2.39	-0.39	-0.55
2019 Rep1	29.13–44.41	36.66	3.43	-0.40	-0.45
2019 Rep2	27.81–45.23	37.07	3.43	-0.67	0.08
2019 Avg.	29.82–44.82	36.87	3.31	-0.48	-0.43
2020 Rep1	29.48–41.45	36.50	2.78	-0.53	-0.46
2020 Rep2	31.09–42.35	36.93	2.60	-0.33	-0.72
2020 Avg.	31.06–41.70	36.72	2.60	-0.44	-0.75
2021 Rep1	28.71–40.88	35.04	2.65	-0.30	-0.53
2021 Rep2	26.84–39.10	34.65	2.62	-0.68	0.11
2021 Avg.	27.77–39.94	34.85	2.51	-0.53	-0.14
PC* 2020 Rep1	29.35–46.59	39.30	4.04	-0.54	-0.59
PC* 2020 Rep2	31.51–46.28	39.33	3.67	-0.40	-0.69
PC* 2020 Avg.	31.12–46.02	39.33	3.78	-0.48	-0.67

*PC-protein combustion data from Eurofins Scientific Inc., Des Moines, Iowa

**Table 2:** Descriptive statistics of the 12 oil content data sets for PI 507429 x PI 399084 population.

Variable	Range	Mean	Std Dev	Skewness	Kurtosis
2018 Rep1	13.80–19.95	16.30	1.34	0.26	-0.25
2018 Rep2	12.80–20.34	16.08	1.38	0.45	0.36
2018 Avg.	13.56–20.15	16.19	1.28	0.39	0.12
2019 Rep1	11.61–19.29	15.51	1.85	-0.04	-0.41
2019 Rep2	11.09–19.16	15.19	1.81	-0.01	-0.37
2019 Avg.	11.62–18.96	15.35	1.75	-0.07	-0.47
2020 Rep1	13.10–20.74	16.26	1.63	0.05	-0.36
2020 Rep2	12.33–19.14	15.86	1.50	0.05	-0.42
2020 Avg.	13.10–19.67	16.07	1.49	0.09	-0.45
2021 Rep1	12.60–19.98	16.49	1.56	-0.15	-0.39
2021 Rep2	13.19–19.28	16.59	1.37	-0.13	-0.28
2021 Avg.	13.08–19.34	16.54	1.39	-0.13	-0.33

The broad sense heritability estimates for protein and oil traits in this population were 0.90 and 0.88, respectively, which are very similar to those reported earlier by Hyten et al. [43], Eskandari et al. [11], Mao et al. [44], Wang et al. [18], and Zhang et al. [45]. Pearson’s correlation between protein and oil content traits for the years 2018–2021 for both replications and the average of those replications for each year were strongly negative (Table 3). This very strong negative correlation, up to -0.87, between protein and oil content is what would be expected since these traits are a well-known subject in the search for finding a positive correlation for these important soybean seed traits [46].

**Table 3:** Correlations between the protein and oil content traits (%) within the 2018–2021 replications of the RIL population.

	**2018 Rep1 Protein**	**2018 Rep2 Protein**	**2018 Avg. Protein**
**2018 Rep1 Oil**	-0.709	-0.601	-0.702
**2018 Rep2 Oil**	-0.537	-0.749	-0.677
**2018 Avg. Oil**	-0.669	-0.722	-0.740
	**2019 Rep1 Protein**	**2019 Rep2 Protein**	**2019 Avg. Protein**
**2019 Rep1 Oil**	-0.873	-0.728	-0.830
**2019 Rep2 Oil**	-0.743	-0.871	-0.837
**2019 Avg. Oil**	-0.844	-0.834	-0.871
	**2020 Rep1 Protein**	**2020 Rep2 Protein**	**2020 Avg. Protein**
**2020 Rep1 Oil**	-0.812	-0.710	-0.783
**2020 Rep2 Oil**	-0.684	-0.760	-0.742
**2020 Avg. Oil**	-0.787	-0.769	-0.801
	**2021 Rep1 Protein**	**2021 Rep2 Protein**	**2021 Avg. Protein**
**2021 Rep1 Oil**	-0.809	-0.631	-0.757
**2021 Rep2 Oil**	-0.691	-0.770	-0.767
**2021 Avg. Oil**	-0.796	-0.736	-0.805

### Chromosomal map construction and quantitative trait locus identification

The JoinMap 4.0 [41] software package was used for map construction. A total of 12,761 markers including 10,526 SNP loci from GBS, 2,217 SNP markers from SoySNP6k Illumina Beadchip, 26 SSR and one indel type marker were mapped on 20 linkage groups using DNA from F_7_ RILs (S2 Table). MapQTL 5.0 [42] was used for QTL identification, and resulted in the identification of two QTLs. A large effect QTL was identified on chromosome 2 and another on chromosome 15.

**Fig 1:**
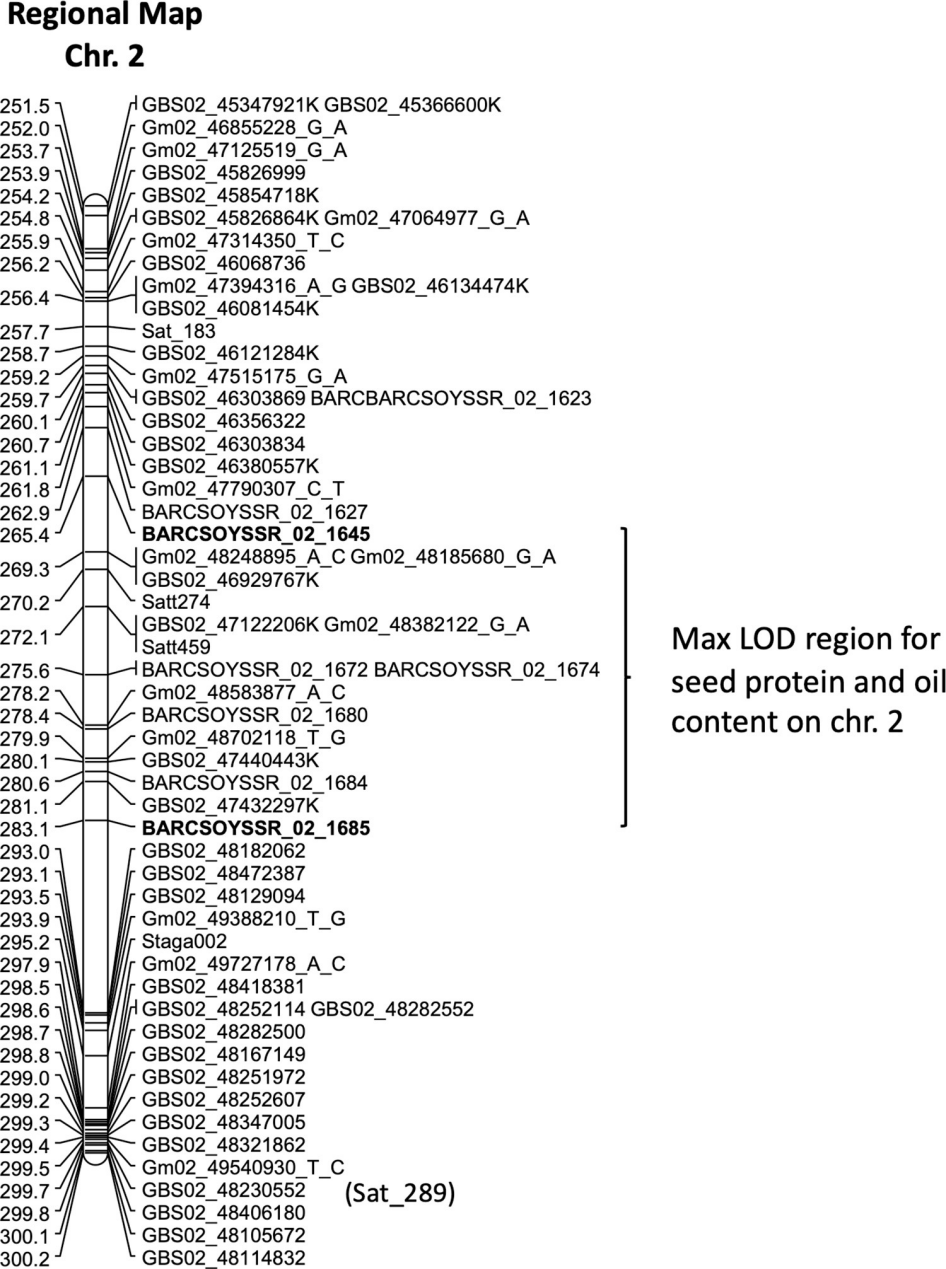
A regional map of chromosome 2 of the PI 507429 x PI 399084 RIL population showing the maximum LOD region of the major seed protein and oil QTL detected between 265.4–283.1 cM. The set LOD threshold was 3.5. (Markers ending in K are GBS SNP markers converted to a KASP marker).

The maximum LOD scores for the major seed protein QTL on chromosome 2 using NIRS data ranged from a low of 4.59 for replication 2 in 2018 to a high of 17.48 for replication 1 of the 2020 protein combustion data (Table 4, column 3). The level of phenotypic variation of the major QTL on chromosome 2, as shown in Table 4 (column 4), ranged from a minimum of 20.1% to a maximum of 56.8%. The QTL region between markers flanking the max LOD points was variable. However, depending on the data set, the map position at the maximum LOD varied only from 279 to 281 cM except for the 2019 data where replication 2 had a position of 274.1 cM (Table 4, column 2). Markers at or closest to the max LOD points are shown in Table 4, column 5. Two markers BARCSOYSSR_02_1645 and BARCSOYSSR_02_1685 flank the maximum LOD (Table 4, column 3) regions resulting from each of the 15 data sets. The regional map for the QTL along chromosome 2 can be seen in Fig 1.

**Table 4:** Quantitative trait locus detected on chromosome 2 using composite interval mapping in the RIL population PI 507429 x PI 399084 using NIRS data sets from 2018–2021 and protein combustion using seeds from the 2020 harvest.

Year/Trait/Replication	Map position at max LOD, cM	Max LOD value	Phenotypic variation, %	Marker at or closest to the max LOD point	Markers flanking max LOD region	Map range (cM) between markers flanking max LOD region
**Seed Protein Content**						
2018 Protein Rep. 1	281.1	7.84	31.4	GBS02_47432297K^+^	BARCSOYSSR_02_1645-BARCSOYSSR_02_1685	265.4–283.1
2018 Protein Rep. 2	281.1	4.59	20.1	GBS02_47432297K^+^	BARCSOYSSR_02_1645-BARCSOYSSR_02_1685	265.4–283.1
2018 Protein Avg.	281.1	7.84	31.4	GBS02_47432297K^+^	BARCSOYSSR_02_1685-BARCSOYSSR_02_1685	265.4–283.1
2019 Protein Rep. 1	280.1	11.57	42.6	GBS02_47440443K^+^	BARCSOYSSR_02_1645-BARCSOYSSR_02_1685	265.4–283.1
2019 Protein Rep. 2	274.1	13.71	50.6	Satt459	BARCSOYSSR_02_1645-BARCSOYSSR_02_1685	265.4–283.1
2019 Protein Avg.	274.1	13.72	50.8	Satt459	BARCSOYSSR_02_1645-BARCSOYSSR_02_1685	265.4–283.1
2020 Protein Rep. 1	280.1	14.58	50.3	GBS02_47440443K^+^	BARCSOYSSR_02_1645-BARCSOYSSR_02_1685	265.4–283.1
2020 Protein Rep. 2	280.1	13.78	48.7	GBS02_47440443K^+^	BARCSOYSSR_02_1645-BARCSOYSSR_02_1685	265.4–283.1
2020 Protein Avg.	280.1	15.42	52.3	GBS02_47440443K^+^	BARCSOYSSR_02_1645-BARCSOYSSR_02_1685	265.4–283.1
Protein Combustion 2020 Protein Rep. 1	280.1	17.48	56.8	GBS02_47440443K^+^	BARCSOYSSR_02_1685-BARCSOYSSR_02_1685	265.4–283.1
Protein Combustion 2020 Protein Rep. 2	280.1	14.94	51.5	GBS02_47440443K^+^	BARCSOYSSR_02_1685-BARCSOYSSR_02_1685	265.4–283.1
Protein Combustion 2020 Protein Avg.	280.1	17.20	56.2	GBS02_47440443K^+^	BARCSOYSSR_02_1685-BARCSOYSSR_02_1685	265.4–283.1
2021 Protein Rep. 1	280.1	8.89	34.7	GBS02_47440443K^+^	BARCSOYSSR_02_1685-BARCSOYSSR_02_1685	265.4–283.1
2021 Protein Rep. 2	279.4	12.66	45.7	Gm02_48702118_T_G	BARCSOYSSR_02_1685-BARCSOYSSR_02_1685	265.4–283.1
2021 Protein Avg.	280.1	12.03	43.9	GBS02_47440443K^+^	BARCSOYSSR_02_1685-BARCSOYSSR_02_1685	265.4–283.1
**Seed Oil Content**						
2018 Oil Rep. 1	275.6	4.1	17.9	BARCSOYSSR_02_1672	Gm02_48248895_A_C-BARCSOYSSR_02_1684	269.3–280.1
2018 Oil Rep. 2	275.6*	3.1*	14.0*	BARCSOYSSR_02_1672*		
2018 Oil Avg.	275.6	4.34	18.8	BARCSOYSSR_02_1672	Gm02_48248895_A_C-BARCSOYSSR_02_1684	269.3–280.1
2019 Oil Rep. 1	276.6	9.74	38.7	BARCSOYSSR_02_1672	BARCSOYSSR_02_1645-BARCSOYSSR_02_1685	265.4–283.1
2019 Oil Rep. 2	276.6	10.21	40.1	BARCSOYSSR_02_1672	BARCSOYSSR_02_1645-BARCSOYSSR_02_1685	265.4–283.1
2019 Oil Avg.	276.6	11.20	43.0	BARCSOYSSR_02_1672	BARCSOYSSR_02_1645-BARCSOYSSR_02_1685	265.4–283.1
2020 Oil Rep. 1	279.4	8.61	34.7	Gm02_48702118_T_G	BARCSOYSSR_02_1645-BARCSOYSSR_02_1685	265.4–283.1
2020 Oil Rep. 2	280.1	7.60	30.8	GBS02_47440443K^+^	BARCSOYSSR_02_1645-BARCSOYSSR_02_1685	265.4–283.1
2020 Oil Avg.	279.4	8.94	35.6	Gm02_48702118_T_G	BARCSOYSSR_02_1645-BARCSOYSSR_02_1685	265.4–283.1
2021 Oil Rep. 1	276.6	5.01	22.4	BARCSOYSSR_02_1672	Satt459- BARCSOYSSR_02_1684	272.1–280.1
2021 Oil Rep. 2	276.6	6.83	28.6	BARCSOYSSR_02_1672	BARCSOYSSR_02_1645-BARCSOYSSR_02_1685	265.4–283.1
2021 Oil Avg.	276.6	6.60	28.1	BARCSOYSSR_02_1672	Satt459- BARCSOYSSR_02_1685	272.1–283.1

^+^GBS SNP marker converted to KASP marker; *Below set LOD threshold of 3.5

The seed protein QTL region on chromosome 2 was also associated with the QTL for seed oil content with a LOD score varying from 4.1 to 11.2 (Table 4, column 3). The map positions at the maximum LOD ranged from 275.6 cM for the 2018 data set of seed oil content to 280.1 cM for the 2021 data (Table 4, column 2). These maximum LOD map positions for seed oil content fall within the seed protein content maximum LOD map locations (Table 4 column 2). The level of explained phenotypic variation for oil content had a minimum of 17.9% based on the 2018 data set and a maximum of 43% in the 2019 data set (Table 4, column 4). The regional map for the chromosome 2 seed protein and oil QTL region can be seen in Fig 1.

**Fig 2:**
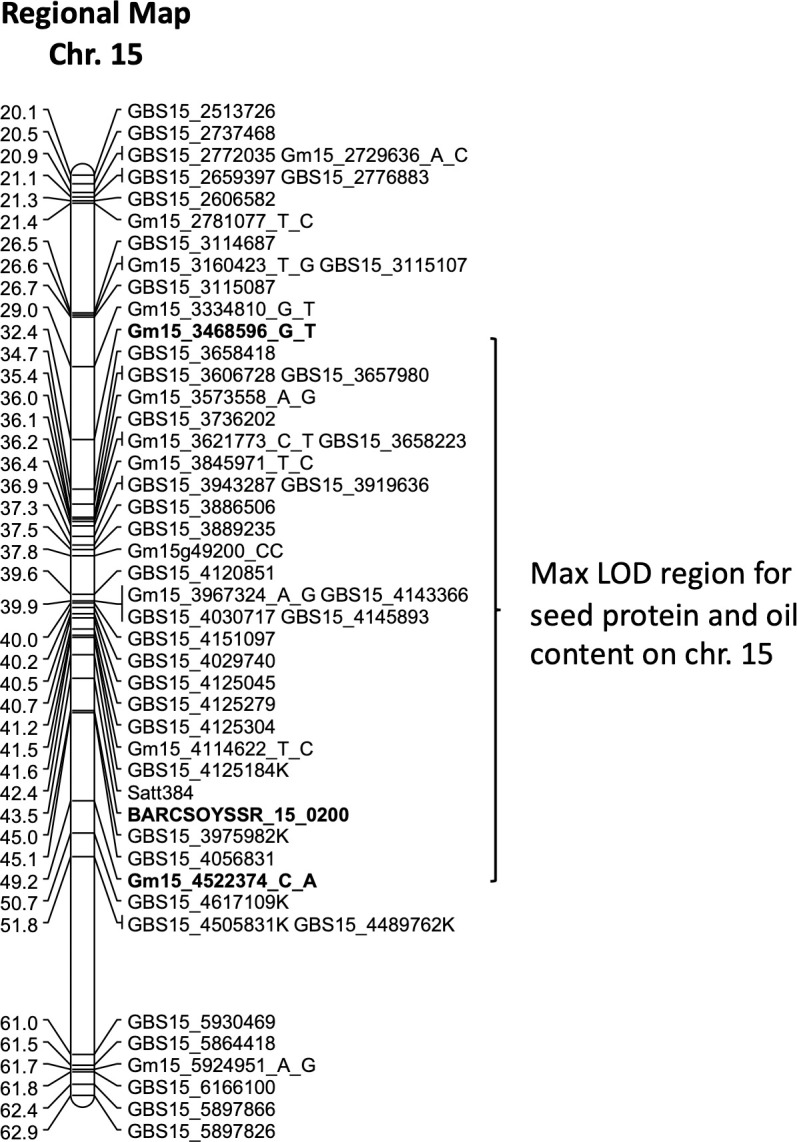
A regional map of chromosome 15 of the PI 507429 x PI 399084 RIL population showing the maximum LOD region of the seed protein and oil QTL detected between 32.4 to 49.2 cM. SSR marker Sat_289, a marker used in previous studies, was monomorphic in our population. Therefore, its expected location is shown in parentheses. The set LOD threshold was 3.5. (Those markers ending in K are GBS SNP markers converted to a KASP marker).

A QTL for seed protein content was also identified on chromosome 15. The minimum and maximum explained phenotypic variation for these data sets (2018–2021) were 16% and 27.2%, respectively (Table 5, column 4). Depending on the data set, map positions at max LOD varied from 37.5 to 39.9 cM (Table 5, column 2). The chromosomal region between markers flanking the max LOD map positions extended from 32.4 cM (Gm15_3468596_G_T) to 43.5 cM (BARCSOYSSR_15_0200) (Table 5, last column, Fig 2). This protein QTL region also appears to be associated with seed oil content. The genetic map positions corresponding to maximum LOD scores for the seed oil content trait were identified within the range of 32.4 to 49.2 cM (Table 5, last column) designated by SNPs Gm15_3468596_G_T and Gm15_4522374_C_A (Fig 2). The minimum and maximum percent explained phenotypic variation of the seed oil data sets were 21% and 41%, respectively.

**Table 5:** Quantitative trait locus detected on chromosome 15 using composite interval mapping in the RIL population, PI 507429 x PI 399084 using NIRS data sets from 2018–2021 and protein combustion using seeds from the 2020 harvest.

Year/Trait/Replication	Map position at max LOD, cM	Max LOD value	Phenotypic variation, %	Marker at or closest to the max LOD point	Markers flanking max LOD region	Map range (cM) between markers flanking max LOD region
**Seed Protein Content**						
2018 Protein Rep. 1	39.9	4.64	20.3	Gm15_3967324_A_G	GBS15_3943287- Satt384	36.9–42.4
2018 Protein Rep. 2	39.9	4.0	18.2	Gm15_3967324_A_G	GBS15_4120851-GBS15_4125279	38.8–40.7
2018 Protein Avg.	39.9	5.0	21.7	Gm15_3967324_A_G	GBS15_3943287- Satt384	36.9–42.4
2019 Protein Rep. 1	37.8	5.5	23.2	Gm15g49200_CC	Gm15_3573558_A_G-BARCSOYSSR_15_0200	36.0–43.5
2019 Protein Rep. 2	37.5	4.86	20.9	GBS15_3889235	Gm15_3573558_A_G-BARCSOYSSR_15_0200	36.0–43.5
2019 Protein Avg.	37.5	5.61	23.8	GBS15_3889235	Gm15_3573558_A_G -BARCSOYSSR_15_0200	36.0–43.5
2020 Protein Rep. 1	37.8*	3.1*	14.8*	Gm15g49200_CC *	*	*
2020 Protein Rep. 2	39.6	4.66	20.9	GBS15_4120851	Gm15_3845971_T_C-Satt384	36.4–42.4
2020 Protein Avg.	39.6	4.09	18.6	GBS15_4120851	GBS15_3886506-Gm15_4114622_T_C	37.3–41.5
Protein Combustion 2020 Protein Rep. 1	38.8	3.52	16.0	Gm15g49200_CC	*	*
Protein Combustion 2020 Protein Rep. 2	38.8	4.2	19.4	Gm15g49200_CC	GBS15_3886506- GBS15_4125045	34.7–40.5
Protein Combustion 2020 Protein Avg.	38.8	3.93	17.9	Gm15g49200_CC	GBS15_3886506-GBS15_4125045	34.7–40.5
2021 Protein Rep. 1	38.8	6.22	27.2	Gm15g49200_CC	Gm15_3468596_G_T-BARCSOYSSR_15_0200	32.4–43.5
2021 Protein Rep. 2	37.8	3.87	16.9	Gm15g49200_CC	Gm15_3845971_T_C-GBS15_4120851	36.4–39.6
2021 Protein Avg.	37.5	5.42	23.1	GBS15_3889235	Gm15_3468596_G_T-BARCSOYSSR_15_0200	32.4–43.5
**Seed Oil Content**						
2018 Oil Rep. 1	41.1	8.63	35.1	GBS15_4125304	Gm15_346596_G_T-Gm15_4522374_C_A	32.4–49.2
2018 Oil Rep. 2	39.9	5.97	25.7	GBS15_4145893	Gm15_346596_G_T-Gm15_4522374_C_A	32.4–49.2
2018 Oil Avg.	42.4	8.57	33.7	Satt384	Gm15_346596_G_T-Gm15_4522374_C_A	32.4–49.2
2019 Oil Rep. 1	36.9	6.72	27.6	GBS15_3943287	Gm15_346596_G_T-BARCSOYSSR_15_0200	32.4–43.5
2019 Oil Rep. 2	36.9	7.03	28.6	GBS15_3943287	Gm15_346596_G_T-BARCSOYSSR_15_0200	32.4–43.5
2019 Oil Avg.	36.9	7.62	30.6	GBS15_3943287	Gm15_346596_G_T-BARCSOYSSR_15_0200	32.4–43.5
2020 Oil Rep. 1	42.4	4.88	21.0	Satt384	Gm15_3967324_A_G BARCSOYSSR_15_0200	39.9–43.5
2020 Oil Rep. 2	38.8	7.27	29.7	Gm15g49200_CC	Gm15_3845971_T_C T-BARCSOYSSR_15_0200	36.4–43.5
2020 Oil Avg.	43.4	6.40	26.5	BARCSOYSSR_15_0200	Gm15_3845971_T_C-BARCSOYSSR_15_0200	36.4–43.5
2021 Oil Rep. 1	38.8	10.12	40.5	Gm15g49200_CC	Gm15_346596_G_T-Gm15_4522374_C_A	32.4–49.2
2021 Oil Rep. 2	36.9	8.24	32.8	GBS15_3943287	Gm15_346596_G_T-Gm15_4522374_C_A	32.4–49.2
2021 Oil Avg.	38.8	10.40	41.0	Gm15g49200_CC	Gm15_346596_G_T-Gm15_4522374_C_A	32.4–49.2

* Below set LOD threshold of 3.5

### Effect of CC indel on protein/oil content

Zhang et al. [26] identified a CC deletion (CC-) in the coding sequence of *Glyma*.*15G049200* (*GmSWEET39*, a sugar transporter), that caused a reading frameshift. This gene located on soybean chromosome 15 was associated with both oil and protein content in the accessions they studied. Since the parents of our population were polymorphic for this indel (PI 507429 CC-; PI 399084 CC+), it was incorporated into the chromosome 15 genetic map and used for protein/oil content QTL mapping in this study. The genetic map position of the CC indel locus as well as its orientation relative to surrounding SNP loci were in good agreement with the reference physical map of Williams 82. Our mapping confirmed a close association of the CC indel marker with the detected protein and oil content QTLs on chromosome 15 in this study (Fig 2, Table 5 column 5).

Zhang et al. [26] proposed that the two distinct alleles of *GmSWEET39* have had two separate avenues for the improvement process. The CC- allele has been inadvertently selected during breeding for oil content improvement and the CC+ allele for protein content improvement [26]. In the Zhang et al. [26] study looking at differences between wild, landrace and cultivars, the landrace lines had an average increase of 2.94% in oil in CC- lines with a decrease of 1.15% in protein content and the two parents of this population, PI 507429 and PI 399084, are landrace lines.

## Discussion

Soybean protein and oil content traits are quantitatively inherited. In the current study, the RIL population of PI 507429 x PI 399084 was evaluated in two replications in each of the planting years 2018 to 2021 and seed protein and oil content data were collected. Quantitative trait locus mapping was used and large effect QTLs were detected on chromosomes 2 and 15. The large effect QTL on chromosome 2 explained a high level of phenotypic variation in each replication/year for seed protein up to 56.8% and oil content up to 43%. Similarly, the QTL on chromosome 15 accounted for up to 27.2% of the variation for protein content and up to 41% for oil content.

Protein and oil content QTL have previously been reported on all twenty soybean chromosomes (Soybase.org). The QTL on chromosomes 15 and 20 are frequently identified as having significant effects on protein content. The QTL on chromosome 15 identified in this study continued this trend, showing phenotypic variation of the seed protein content between 16% and 27.2% and phenotypic variation for oil content between 21% and 41%. The QTL detected on chromosome 15 for both traits mapped to a region between flanking markers Gm15_3468596_G_T and Gm15_4522374_C_A (Table 5, Fig 2). This is in the same region that other protein and oil QTLs on chromosome 15 have been identified in other reports [20, 26, 47–51]. The present study also confirmed a close association of the CC indel marker (Gm15g49200_CC) developed on GmSWEET39 gene sequence which was earlier identified by Zhang et al. [26] with detected protein and oil content QTLs on chromosome 15 (Table 5, Fig 2).

The maximum LOD QTL region identified for the protein and oil traits on chromosome 2 was between 265.4 and 283.1 cM including the Satt459 SSR locus (Fig 1). Hyten et al. [43] were the first to report a protein QTL on chromosome 2, which was associated with the Satt459 marker. The same region was later reported by Qi et al. [52] to contain an oil QTL. Wang et al. [18] also mapped a protein QTL between SSR markers Satt274 and Sat_289 which encompasses the Satt459 region (Fig 1). The seed oil QTL was also mapped with markers Satt274 and Satt459 on chromosome 2 by others [52–54]. In these reports the physical position of the protein and oil QTL falls into an interval of 45.3 to 47.0 Mbp (Glyma 2.0) between SSR loci Satt274 and Sat_289, which almost coincides with the starting point of the QTL interval identified in our study but extends about 1.3 Mbp further down to Sat_289 region. Our results confirmed the seed protein and oil QTL location on chromosome 2. In this study, we were able to narrow down the protein and oil QTL region on chromosome 2 from about 1.7 to 0.8 Mbp. This calculation is based on physical distance comparison between previously reported QTL markers Satt274 and Sat_289 (45,267,222 to 47,042,650 bp) and those identified in this study BARCSOYSSR_02_1645 and BARCSOYSSR_02_1685 (44,939,870 to 45,728,856 bp). This was made possible by incorporating numerous SNP markers for genetic map construction. Also, the max LOD scores and the corresponding levels of explainable phenotypic variation (Table 4) allowed us to claim high significance status for the protein and oil QTL on chromosome 2 identified in this study.

Previous studies have identified minor effect seed protein and oil QTLs in other regions of the chromosome 2 as in the report by Gillenwater et al. [55] who identified four minor effect QTL: one for protein, one for oil and two for both seed protein and oil content each explaining less than 10% of the phenotypic variation. Kabelka et al. [56] identified a QTL on LG D1b (chromosome 2) that explained 14% of the phenotypic variation for protein content. Chen et al. [49] identified a minor QTL on LG D1b explaining 5.16% for protein located near Sat_135 and Satt537. Mao et al. [44] observed a QTL on chromosome 2 that explained 29% of the phenotypic variation for protein content. This group also observed two other QTL on chromosome 2 but all had minor effect. Qi et al. [57] also observed this same QTL that Mao et al. [44] identified but in their study, it explained 10.92% of the phenotypic variation and in only one environment. Wang et al. [18] identified a protein content QTL on chromosome 2 that explained between 12.3% and 16.4% of the phenotypic variation. The present study detected a major effect QTL on chromosome 2, explaining up to 56.8% and 43% of the variation for protein and oil content, respectively.

Variability from year to year in seed protein and oil content in this population may partially be attributed to the environmental conditions during seed filling. The RIL population described herein was grown only in one location but was observed over four years with 2 replications each and the temperature and water availability conditions varied each year. Previous studies have observed mixed results of environmental factors such as temperature and water availability [15, 58, 59]. In our study, in years where the temperature was above average during seed fill and water was limited such as in 2019, we noticed a reduction in protein content whereas, in 2018, where the opposite was observed, the protein content was noticeably higher for some lines.

We observed a high heritability of protein and oil traits in this population that was consistent with the values reported by others [11, 18, 43, 45, 60]. The high heritability of these two traits would indicate that a considerable amount of the variation within the population is genetic. We also observed a negative correlation between seed protein and oil content traits for the years 2018–2021 for both replications and the average of those replications. This negative correlation between protein and oil content is what would be expected since these traits are known to be negatively correlated and reported in other mapping populations [13, 18, 43, 50, 61].

In this study, the two QTL identified on chromosomes 2 and 15 appear to be strongly associated with both seed protein and oil contents. Earlier studies such as Pathan et al. [61] identified two QTL for protein and oil on chromosomes 5 and 6. Hwang et al. [62] identified three QTL regions marked by seven SNP loci on chromosomes 8, 9 and 20 associated with both seed protein and oil content. Bandillo et al. [19] identified multiple SNPs on chromosomes 15 and 20 through GWAS that were associated with both oil and protein contents. Seo et al. [63] identified four QTL for both traits in a selected breeding population. Zhu et al. [64] detected QTL controlling both seed protein and oil content on chromosomes 8, 15 and 20.

Considering the high broad-sense heritability found within this population, selection for the seed protein and oil content seems possible. This population contains lines that have protein content values higher than PI 399084 and other lines have oil content values higher than PI 507429. These lines and the molecular markers identified in this study may be useful in a breeding program when selecting for increased seed protein or oil content. Fifteen of the SNPs mapping to chromosomes 2 and 15 were converted to KASPR markers facilitating their use in breeding programs.

## Conclusion

In this study, using a RIL population, we constructed a high-resolution map, analyzed protein and oil content data from 4 years of field testing and identified large effect QTLs on chromosomes 2 and 15 for these traits. Protein content QTL mapping results based on NIRS assay was confirmed using protein combustion data. The protein and oil QTLs identified in this study were compared to those previously detected on these same chromosomes. The genetic materials of this study based on two plant introductions with wider protein and oil contents resulted in the identification of a QTL on chromosome 2 accounting for up to 56% of variation for protein and 43% for oil content, which are larger than those from previous studies. Furthermore, the QTL region was narrowed down from 1.7 to 0.8 Mbp. The QTL on chromosome 15 was identified in the same region as previously verified QTLs on this chromosome, it accounted for up to 27.2% of variation for protein and up to 41% of variation for oil content.

## Supporting information

S1 Table Primer sequences of GBS SNP markers converted to KASP. (DOCX)

S2 Table The type and the number of markers per chromosomes used for QTL mapping in the RIL population of PI 507429 x PI 399084. (DOCX)

S3 Table 2018–2021 Seed protein and oil data. (XLSX)

S4 Table Genotypic data for chromosomes 2 and 15. (XLSX)
