# Supplementary material for: Identification of quantitative trait loci controlling soybean seed protein and oil content
Source: PLoS One. 2023 Jun 23;18(6):e0286329. doi: 10.1371/journal.pone.0286329 (PMC10289428; doi:10.1371/journal.pone.0286329)
Supplement: S1 Table — (DOCX) [file pone.0286329.s001.docx]

| **Supplementary Table 1. Primer sequences of GBS SNP markers converted to KASP.** | | |
| --- | --- | --- |
| **KASP-GBS SNP marker ID** | **Oligo function** | **Oligo sequence 5'-3'** |
| GBS02_45347921K | Allele specific oligo 1 | GAA GGT GAC CAA GTT CAT GCA TAA CGA CAA AAG ATT GTT TAG |
|  | Allele specific oligo 2 | GAA GGT CGG AGT CAA CGG ATT ATA ACG ACA AAA GAT TGT TTA C |
|  | Common primer | CTT GTT GAT GGA TAT GTA TAA TTG A |
| GBS02_45366600K | Allele specific oligo 1 | GAA GGT GAC CAA GTT CAT GCA AGT TAC AAA GAT CAC TCA AGT |
|  | Allele specific oligo 2 | GAA GGT CGG AGT CAA CGG ATT AAG TTA CAA AGA TCA CTC AAG C |
|  | Common primer | TGT TCA TTT AAA ACA AGA TTT AGT T |
| GBS02_45826864K | Allele specific oligo 1 | GAA GGT GAC CAA GTT CAT GCA GGC GAA GAG CTT CAT AAT |
|  | Allele specific oligo 2 | GAA GGT CGG AGT CAA CGG ATT AGG CGA AGA GCT TCA TAA C |
|  | Common primer | AGC ATG CGG CGA GAA GAG GC |
| GBS02_45854718K | Allele specific oligo 1 | GAA GGT GAC CAA GTT CAT GCT TCT ACA TAT AAA TAG GGC AC |
|  | Allele specific oligo 2 | GAA GGT CGG AGT CAA CGG ATT CTA CAT ATA AAT AGG GCA T |
|  | Common primer | AGT ATA TTC CCC GAA GCA AT |
| GBS02_46081454K | Allele specific oligo 1 | GAA GGT GAC CAA GTT CAT GCA ATA GCA ATA GCT GCA GCT |
|  | Allele specific oligo 2 | GAA GGT CGG AGT CAA CGG ATT AAT AGC AAT AGC TGC AGC G |
|  | Common primer | TTG GGT GGG AAT CCA CCA ACT |
| GBS02_46121284K | Allele specific oligo 1 | GAA GGT GAC CAA GTT CAT GCT GTT TCT TTG CAC GGC CAC T |
|  | Allele specific oligo 2 | GAA GGT CGG AGT CAA CGG ATT TGT TTC TTT GCA CGG CCA CC |
|  | Common primer | TCA ACT GCA GCT CCC TGC ATG |
| GBS02_46134474K | Allele specific oligo 1 | GAA GGT GAC CAA GTT CAT GCA GTT TTT GAG TTG TTT TTC TCT TCG |
|  | Allele specific oligo 2 | GAA GGT CGG AGT CAA CGG ATT AGT TTT TGA GTT GTT TTT CTC TTC C |
|  | Common primer | CAT TCT ACA ATA TTT TAT CCA ACA AA |
| GBS02_46380557K | Allele specific oligo 1 | GAA GGT GAC CAA GTT CAT GCT GTA CAA AAT AAA GCA TAA AGT |
|  | Allele specific oligo 2 | GAA GGT CGG AGT CAA CGG ATT GTA CAA AAT AAA GCA TAA AGC |
|  | Common primer | CTT TGA ACC ATT CTA CAC TTC |
| GBS02_46929767K | Allele specific oligo 1 | GAA GGT GAC CAA GTT CAT GCA ATA TCT AGG GGT GCT CGC |
|  | Allele specific oligo 2 | GAA GGT CGG AGT CAA CGG ATT AAT ATC TAG GGG TGC TCG T |
|  | Common primer | ACT GCT GGA ACT CCT TCA ATC |
| GBS02_47122206K | Allele specific oligo 1 | GAA GGT GAC CAA GTT CAT GCT TCA GTT TCC GTT TGA GTA |
|  | Allele specific oligo 2 | GAA GGT CGG AGT CAA CGG ATT TCA GTT TCC GTT TGA GTG |
|  | Common primer | CCC TTT TCT TCA GAA ACA ATA A |
| GBS02_47432297K | Allele specific oligo 1 | GAA GGT GAC CAA GTT CAT GCA AGC AGG CTG TGA GTA TCA |
|  | Allele specific oligo 2 | GAA GGT CGG AGT CAA CGG ATT AAG CAG GCT GTG AGT ATC G |
|  | Common primer | ATT TCA TGA TTG GCT CCC CAA |
| GBS02_47440443K | Allele specific oligo 1 | GAA GGT GAC CAA GTT CAT GCG TGA AGC AGT TAG GCT TTC |
|  | Allele specific oligo 2 | GAA GGT CGG AGT CAA CGG ATT GTG AAG CAG TTA GGC TTT G |
|  | Common primer | TTA GCC TCT TTA CCA AGA CAT |
| GBS15_3975982K | Allele specific oligo 1 | GAA GGT GAC CAA GTT CAT GCA TGC TCC AGC TCC TAC TAG |
|  | Allele specific oligo 2 | GAA GGT CGG AGT CAA CGG ATT ATG CTC CAG CTC CTA CTA C |
|  | Common primer | GGA TAG GTA CGA TGG TTA AT |
| GBS15_4125184K | Allele specific oligo 1 | GAA GGT GAC CAA GTT CAT GCC TCC GCC CTA ACT GAA ACT |
|  | Allele specific oligo 2 | GAA GGT CGG AGT CAA CGG ATT CTC CGC CCT AAC TGA AAC C |
|  | Common primer | AAG TTC GAA AGG AAT GCT ACG |
| GBS15_4489762K | Allele specific oligo 1 | GAA GGT GAC CAA GTT CAT GCA GTA TTA TCT TCA ACA CTT GC |
|  | Allele specific oligo 2 | GAA GGT CGG AGT CAA CGG ATT GTA TTA TCT TCA ACA CTT GT |
|  | Common primer | ATC AAA TAT TTG ATT TGA CTC AC |
| GBS15_4505831K | Allele specific oligo 1 | GAA GGT GAC CAA GTT CAT GCT AGT AAT TAA TCG ATC CTC G |
|  | Allele specific oligo 2 | GAA GGT CGG AGT CAA CGG ATT AGT AAT TAA TCG ATC CTC A |
|  | Common primer | AGT ATT GGT CCT GCA GTA GCT |
| GBS15_4617109K | Allele specific oligo 1 | GAA GGT GAC CAA GTT CAT GCA TCT CCA GAG ATC AGC CTC CGT |
|  | Allele specific oligo 2 | GAA GGT CGG AGT CAA CGG ATT TCT CCA GAG ATC AGC CTC CGC |
|  | Common primer | TTA GGC ATT TAG CAA ACA AC |

**S1 Table.** Primer sequences of GBS SNP markers converted to KASP.
