# Supplementary material for: Identification of quantitative trait loci controlling soybean seed protein and oil content
Source: PLoS One. 2023 Jun 23;18(6):e0286329. doi: 10.1371/journal.pone.0286329 (PMC10289428; doi:10.1371/journal.pone.0286329)
Supplement: S2 Table — (DOCX) [file pone.0286329.s002.docx]

| **Chromosome** | **GBS SNPs** | **6K Gm-SNPs** | **Total** | **Number of Marker Bins**** |
| --- | --- | --- | --- | --- |
| 1 | 348 | 90 | 438 | 324 |
| 2 | 595 | 135 | 757 | 543 |
| 3 | 493 | 115 | 610 | 438 |
| 4 | 566 | 93 | 659 | 492 |
| 5 | 376 | 102 | 478 | 323 |
| 6 | 690 | 111 | 802 | 603 |
| 7 | 409 | 110 | 519 | 334 |
| 8 | 682 | 144 | 826 | 559 |
| 9 | 407 | 105 | 512 | 344 |
| 10 | 386 | 109 | 495 | 385 |
| 11 | 277 | 99 | 377 | 224 |
| 12 | 317 | 100 | 417 | 276 |
| 13 | 580 | 144 | 726 | 512 |
| 14 | 554 | 102 | 656 | 428 |
| 15 | 935 | 129 | 1,068 | 563 |
| 16 | 501 | 87 | 589 | 425 |
| 17 | 626 | 116 | 744 | 573 |
| 18 | 1,013 | 132 | 1,145 | 928 |
| 19 | 411 | 102 | 513 | 398 |
| 20 | 340 | 90 | 430 | 326 |
| Total: | 10,509 | 2,217 | 12,761* | 8,998 |

*This total also includes 17 GBS-KASP type markers, 26 SSR markers and 1 Indel marker

**Number of markers with unique segregation pattern

**S2 Table.** The type and the number of markers per chromosomes used for QTL mapping in the RIL population of PI 507429 x PI 399084.
